# Potential Causal Association between Plasma Metabolites, Immunophenotypes, and Female Reproductive Disorders: A Two-Sample Mendelian Randomization Analysis

**DOI:** 10.3390/biom14010116

**Published:** 2024-01-16

**Authors:** Hui-Hui Shen, Yang-Yang Zhang, Xuan-Yu Wang, Cheng-Jie Wang, Ying Wang, Jiang-Feng Ye, Ming-Qing Li

**Affiliations:** 1 Laboratory for Reproductive Immunology, Hospital of Obstetrics and Gynecology, Fudan University, Shanghai 200080, China; 2 Shanghai Medical College, Fudan University, Shanghai 200032, China; 3 College of Traditional Chinese Medicine, Tianjin University of Traditional Chinese Medicine, No. 10, Poyang Lake Road, Tuanpo Xinchengxi District, Jinghai District, Tianjin 301617, China; 4 Department of Obstetrics and Gynecology, Hospital of Obstetrics and Gynecology, Fudan University, Shanghai 200011, China; 5 Center for Reproductive Medicine, Department of Obstetrics and Gynecology, Qilu Hospital, Shandong University, Ji’nan 250012, China; 6 Institute for Molecular and Cell Biology, Agency for Science, Technology and Research, Singapore 138632, Singapore; 7 Shanghai Key Laboratory of Female Reproductive Endocrine Related Diseases, Hospital of Obstetrics and Gynecology, Fudan University, Shanghai 200080, China

**Keywords:** immunophenotypes, metabolites, gestational diabetes, Mendelian randomization, mannose, single-nucleotide polymorphisms

## Abstract

Background: While extensive research highlighted the involvement of metabolism and immune cells in female reproductive diseases, causality remains unestablished. Methods: Instrumental variables for 486 circulating metabolites (*N* = 7824) and 731 immunophenotypes (*N* = 3757) were derived from a genome-wide association study (GWAS) meta-analysis. FinnGen contributed data on 14 female reproductive disorders. A bidirectional two-sample Mendelian randomization study was performed to determine the relationships between exposures and outcomes. The robustness of results, potential heterogeneity, and horizontal pleiotropy were examined through sensitivity analysis. Results: High levels of mannose were found to be causally associated with increased risks of gestational diabetes (GDM) (OR [95% CI], 6.02 [2.85–12.73], *p* = 2.55 × 10^−6^). A genetically predicted elevation in the relative count of circulating CD28^−^CD25^++^CD8^+^ T cells was causally related to increased female infertility risk (OR [95% CI], 1.26 [1.14–1.40], *p* = 1.07 × 10^−5^), whereas a high absolute count of NKT cells reduced the risk of ectopic pregnancy (OR [95% CI], 0.87 [0.82–0.93], *p* = 5.94 × 10^−6^). These results remained consistent in sensitivity analyses. Conclusions: Our study supports mannose as a promising GDM biomarker and intervention target by integrating metabolomics and genomics.

## 1. Introduction

In the context of global trends of delayed childbearing and declining fertility rates, reproductive health and the challenges related to pregnancy have become significant concerns [1]. However, drug development in this area faces persistent obstacles due to ethical and fetal safety constraints. It is crucial to identify biomarkers for the early detection of individuals at risk of female reproductive diseases. Equally important is the requirement for modifiable biomarkers that can be targeted for preventive or therapeutic interventions.

In recent years, many high-throughput technologies, including genomics, transcriptomics, and metabolomics, have been widely applied in reproductive research. Among them, the metabolome offers highly predictive phenotype information, as it represents the downstream products of interactions among genes, transcripts, proteins, and metabolites. Consequently, blood metabolites serve as practical markers for personalized medicine and disease process monitoring [2,3]. A comprehensive review of the literature on biochemical biomarkers, which included nine metabolomics studies, has highlighted the critical role of histidine metabolism in early miscarriage [4]. In our experimental study, mice administered with fructose-1,6-bisphosphate (FBP) showed a significant increase in plasma and uterine FBP levels [5]. This increased FBP led to the activation of COX-2^+^ decidual macrophages, effectively reducing pregnancy loss by promoting decidualization, trophoblast invasion, and maternal–fetal immune tolerance [5]. A cross-sectional study revealed that pregnancy, as physiological, and polycystic ovary syndrome (PCOS), as a pathological state, mutually hold the risk for developing glucose metabolism irregularity [6]. Gestational diabetes (GDM) is characterized by glucose intolerance during pregnancy, impacting approximately 14.7% of all pregnancies [7]. It not only poses a serious threat to maternal safety, leading to conditions like preeclampsia (PE) or eclampsia, but also poses long-term cardiovascular disease (CVD) and type 2 diabetes (T2D) susceptibility in mothers and offspring [8]. A previous study suggested lysophosphatidylcholine, oleic acid, and 1,25-dihydroxyvitamin D3-26,23-lactones were closely linked with the initiation and development of GDM and PE [9]. However, the causal relationship between plasma metabolites and female reproductive diseases is still pending.

While observational and experimental studies have indicated links between immune dysfunction and various female reproductive diseases such as endometriosis [10], ectopic pregnancy [11], female infertility [12], premature rupture of membranes [13], postpartum hemorrhage [14], and preterm labor and delivery [15], establishing definitive causal associations is challenging due to small sample sizes and limited insights about pathophysiological mechanisms. 

Mendelian randomization (MR) analysis, a method in genetic epidemiology, leverages the extensive data from publicly available large-scale genome-wide association studies (GWASs) to pinpoint genetic variants linked to specific traits or diseases [16]. The fixed nature of genetic variants, which are randomly allocated at conception and relatively unaffected by environmental factors, allows MR to provide an unbiased estimation of causal effects [17]. To identify potential circulating biomarkers of immunity and metabolism in these disorders, we perform a bidirectional two-sample MR analysis to identify causal associations between genetically determined metabolites, immune traits, and 14 female reproductive disorders.

## 2. Material and Methods

### 2.1. Study Design and Ethical Approval

**Figure 1:**
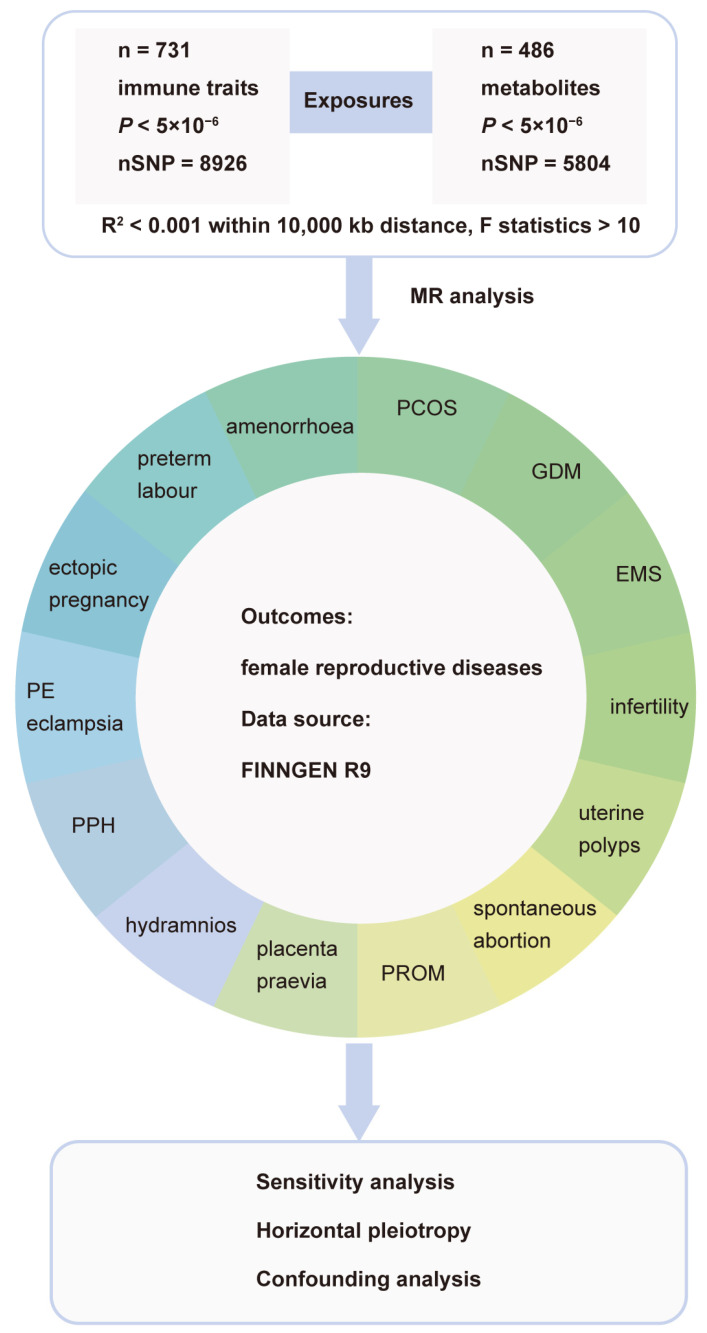
Schematic view of the study design. Abbrev: MR, Mendelian randomization; SNP, single-nucleotide polymorphisms; PCOS, polycystic ovarian syndrome; GDM, gestational diabetes; EMS, endometriosis; PROM, premature rupture of membranes; PPH, postpartum hemorrhage; PE, pre-eclampsia.

An overview of the general design is shown in Figure 1. The study methods were compliant with the Strengthening the Reporting of Observational Studies in Epidemiology–Mendelian Randomization (STROBE-MR) checklist.

FinnGen is a public–private partnership project covering the entire population of Finland, exploring the causes of diverse diseases and promoting population health [18]. It offers summary data for 14 female reproductive illnesses, with relevant GWAS summary statistics presented in Supplementary Table S1. To address potential bias arising from sample overlap, GWAS data for exposure and outcome were sourced from distinct cohorts. All summary statistics utilized in this MR analyses were extracted from previously published studies, for which ethical approval and individual consent were obtained for all original studies.

### 2.2. Genetic Instruments for Metabolites and Immune Traits

Shin et al.’s study provided GWAS summary statistics of serum metabolites [19], which comprised a total of 7824 samples from the German KORA F4 study and British Twins UK cohort [20]. A total of 486 metabolites were selected for our genetic investigation, which could be further divided into eight major categories including amino acids, carbohydrates, cofactors and vitamins, energy, lipids, nucleotides, peptides, and xenobiotic metabolism [21]. A large dataset with over 2.1 million single-nucleotide polymorphisms (SNPs) served as the foundation for the GWAS meta-analysis after stringent quality control. A detailed description of the methods was published previously [22]. 

The GWAS catalog offers public access to GWAS summary statistics for immunological traits (accession numbers GCST90001391 to GCST90002121) [23,24]. There was no subject overlap between cohorts in the initial GWAS on immunological characteristics, which was conducted using data gathered from 3757 European people. After controlling for confounders (i.e., sex and age), relationships were explored for over 22 million SNP genotypes using high-density arrays and imputed using a Sardinian sequence-based reference panel [25].

### 2.3. Instrumental Variables (IVs) Selection

The threshold of the statistically significant level of IVs for every immunophenotype and serum metabolite was adjusted to *p* < 5 × 10^−6^ to identify SNPs strongly associated with the traits of interest due to a limited number of SNPs detected with the threshold at *p* < 5 × 10^−8^ [26]. In MR research, this statistical threshold relaxation was often employed to incorporate a broader set of genetic instruments [27]. Then, we carried out clumping (*R*^2^ < 0.001 within a 10,000 kb window) to obtain independent SNPs based on the 1000 Genomes Project’s linkage disequilibrium (LD) reference panel (https://www.internationalgenome.org/data, accessed on 1 July 2023) [28]. 

Instrumental SNPs were chosen after eliminating palindromic variants with a middle allele frequency (MAF). Due to their low confidence level, SNPs with a MAF less than 0.01 were likewise eliminated from the original GWAS. For each IV, the F statistic and the proportion of explained variance (*R*^2^) were computed in order to assess the effectiveness of the genetic instruments and prevent weak instrumental bias [27,29]. For the purpose of choosing robust instrumental variables, an F statistic of more than 10 was regarded as a common criterion. Any immune traits and serum metabolites with an F statistic of less than 10 were subsequently excluded from the analysis [30].

### 2.4. Statistical Analysis

In our primary analysis, the inverse-variance weighted (IVW) approach was utilized for multiple variants, which predicated on the IVs meeting criteria of relevance, independence, and exclusivity and the assumption that genetic variations affect outcomes solely through the exposure being studied. For a single genetic variant identified, the Wald ratio estimator was adopted. Since there are multiple tests in this study, significant associations between metabolites, immune traits, and 14 female reproductive diseases were identified after adjusting for multiple testing with Bonferroni correction [31]. 

Subsequent validation of these associations was conducted using alternative MR estimation methods (the MR-Egger, weighted median, weighted mode, and simple mode) [32], complemented by heterogeneity and pleiotropy analyses. The ‘Circlize’ R package (v0.4.15) was used to generate Circos plots to compare association analysis data [33]. Due to varied experimental conditions, analytical platforms, and study subjects, heterogeneity might cause bias in causal effect estimates. To address this, Cochran’s Q test was performed to test for heterogeneity [34]. When using IVW for causal analysis, potential confounders and biases due to genetic variability are a concern. To assess pleiotropy, the IVW model and MR-Egger intercept test were applied. An intercept value near 0 (<0.1) and *p* > 0.05 suggests minimal pleiotropy. We further evaluated horizontal pleiotropy and potential outliers using MR-Egger regression and the MR-PRESSO global test using the MR-PRESSO software (v1.0) [35]. Subsequently, we implemented a leave-one-out analysis, a strategy designed to determine whether results were affected by one SNP [36]. Funnel and scatter plots were employed to visually evaluate the symmetry and estimate the effects. 

Additionally, we conducted reverse MR analyses, treating GDM, ectopic pregnancy, and female infertility as exposures and the biomarkers as outcomes. This approach enabled us to assess potential feedback loops between disease risk and biomarker levels, which are crucial for identifying and mitigating false positive results. All MR analyses were carried out using the ‘TwoSampleMR’ [28] R package (v0.5.7). Multivariate MR analysis (MVMR) as a sensitivity analysis to correct for measured confounders was performed using ‘MVMR’ [37] (v0.4) and ‘TwoSampleMR’ [28] R package. 

### 2.5. Colocalization Analyses on Exposure with Outcome

To further validate correlations identified in the MR analyses, we conducted colocalization analyses, employing a Bayesian approach with the ‘coloc’ R package (v5.2.3) [38]. As the reference variant, the variant with the lowest *p*-value and the strongest correlation with the exposure in the MR study was chosen, which includes variants ±1 Mb of the reference SNPs. The study examined five hypotheses that are mutually exclusive: (1) there is no causal SNP for either trait (H0); (2) only trait 1 has a causal SNP (H1); (3) only trait 2 has a causal SNP (H2); (4) both traits have a causal SNP, but the two causal SNPs are distinct (H3); and (5) both traits have a causal SNP and share the same SNP (H4) [39]. A primary focus was directed towards the last hypothesis, H4, and its assessment was based on posterior probability (PP), or PPH4. We identified substantial evidence of colocalization at PPH4 ≥ 0.75 [40] and visualized the colocalization results using the ‘LocusCompareR’ R package (v1.0.0) [41]. All analyses were conducted using R software (version 4.2.3).

### 2.6. Metabolic Pathway Analysis

After MR analysis, the MetaboAnalyst 5.0 software (https://www.metaboanalyst.ca/MetaboAnalyst/faces/home.xhtml, accessed on 1 September 2023) [42] was used to perform a metabolic pathway analysis for the metabolites that were detected by IVW at *p* < 0.05. The underlying metabolite groups or pathways that could be pertinent to the biological process of female reproductive disorders were found using functional enrichment analysis and the pathway analyses module. 

## 3. Results

### 3.1. Strength of the Instrumental Variables (IVs)

This study explored the causal relationships between 731 circulating immunophenotypes, 486 metabolites, and the risk of female reproductive disorders. The 486 metabolite IVs that were selected range in size from 1 to 132 SNPs, while the 731 immunophenotype IVs range from 1 to 93 SNPs. Meanwhile, all IVs were found to be adequately effective for the MR analysis of the 486 metabolites and 731 circulating immunophenotypes (F statistic > 10), as indicated by the minimum F statistic of 19.355 for these IVs. Specifically, the 731 immunophenotypes comprise morphological parameters (MP), relative cell counts (RC), absolute cell counts (AC), and median fluorescence intensities (MFI). Specifically, the MP feature included cDC and TBNK panels, while MFI, AC, and RC encompassed B cells, mature T cell stages, monocytes, myeloid cells, TBNK (T cells, B cells, natural killer cells), and Treg panels. For MR analysis, we employed 1518 SNPs for AC, 4649 SNPs for MFI, 361 SNPs for MP, and 2398 SNPs for RC following the selection and harmonization of IVs. These IVs did not exhibit significant heterogeneity, according to Cochran’s Q test findings.

### 3.2. Identifying the Causal Effect of Immunophenotypes on Female Reproductive Disorders

**Figure 2:**
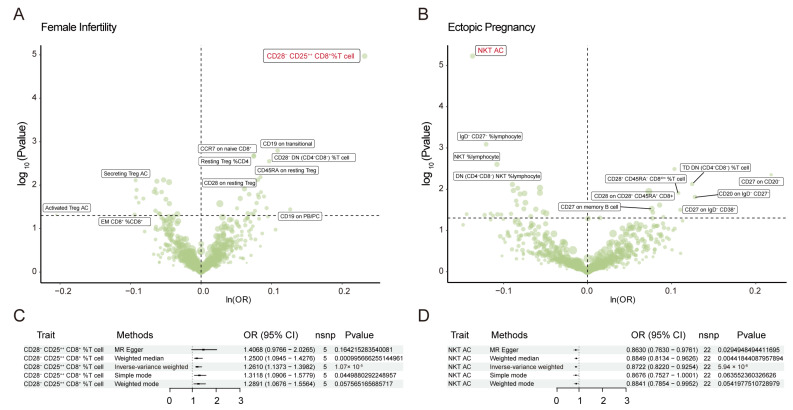
The causal impact of immune traits on female infertility and ectopic pregnancy. (**A**) The volcano plot illustrates the link between 731 immune traits and female infertility risk. The X-axis represents the logarithmic OR (odds ratio) with a base of e, the Y-axis represents the logarithmic *p*-value with a base of 10, and *p*  <  0.05 is considered statistically significant. (**B**) The volcano plot illustrates the link between 731 immune traits and ectopic pregnancy risk. The X-axis represents the logarithmic OR with a base of e, the Y-axis represents the logarithmic *p*-value with a base of 10, and *p* < 0.05 is considered statistically significant. (**C**) The forest plot shows the causal association between CD28^−^CD25^++^CD8^+^ T cell relative count (RC) and female infertility. (**D**) The forest plot shows the causal association between the NKT absolute count (AC) and ectopic pregnancy. OR: odds ratio; CI: confidence interval. NKT: natural killer T cells; OR: odds ratio; SNP, single-nucleotide polymorphisms.

IVW analysis demonstrated a strong causal association between an elevated CD28^−^CD25^++^CD8^+^ T cell RC and an increased risk of female infertility (odds ratio (OR) = 1.26, 95% confidence interval (CI) = 1.14–1.40, *p* = 1.07 × 10^−5^). This association remained statistically significant even after Bonferroni correction (Figure 2A). Additionally, Figure 2B illustrates a potential inverse correlation between a raised natural killer T (NKT) cell AC and an increased probability of ectopic pregnancy (OR = 0.87, 95% CI = 0.82–0.93, *p* = 5.94 × 10^−6^). The most detrimental and protective serum immune trait factors for 14 female reproductive diseases were summarized in Supplementary Table S2.

The outcomes of the weighted mode, weighted median, simple mode, and MR-Egger analysis are shown in Figure 2C,D. Significant outliers were eliminated using the MR-PRESSO in the sensitivity analysis. In addition, there was no discernible heterogeneity according to Cochran’s Q test. Significant horizontal pleiotropy was not found using the ‘leave-one-out’ approach, forest plots, or the MR-Egger intercept test (Supplementary Figures S1 and S2). The results’ stability was demonstrated by the scatter plot (Supplementary Figures S1 and S2).

### 3.3. Identifying the Causal Effect of Metabolites on Female Reproductive Disorders

**Figure 3:**
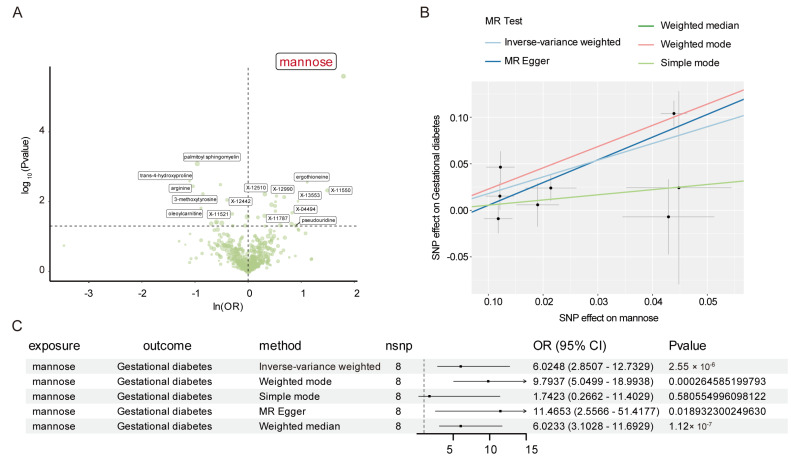
Mendelian randomization (MR) results for the relationship between serum mannose levels and gestational diabetes. (**A**) The volcano plot illustrates the link between 486 metabolite traits and gestational diabetes risk. The X-axis represents the logarithmic OR with a base of e, the Y-axis represents the logarithmic *p*-value with a base of 10, and *p* < 0.05 is considered statistically significant. (**B**) IVW Mendelian randomization estimates, MR-Egger estimates, followed by four additional MR methods (the MR-Egger, weighted median, weighted mode, and simple mode) for the association between mannose and gestational diabetes. IVW, inverse-variance weighted; MR, Mendelian randomization; SNP, single-nucleotide polymorphisms. (**C**) Forest plot shows the causal association between mannose and gestational diabetes using different methods. OR: odds ratio; CI: confidence interval.

Utilizing comprehensive metabolomics data, we explored the causal relationship between genetically predicted metabolic characteristics on the liability of 14 female reproductive disorders. The most detrimental and protective serum metabolite factors for 14 female reproductive diseases were summarized in Supplementary Table S3. Through IVW analysis, our data exhibited that an increased level of mannose had a causal effect on a higher risk of GDM (OR = 6.02, 95% CI = 2.85–12.73, *p* = 2.55 × 10^−6^) (Figure 3A). This relationship remained significant even after Bonferroni adjustment (Figure 3A). Forest plots to visualize the causal effect of every SNP (rs10736, rs11676911, rs12218544, rs12414366, rs1260326, rs13116473, rs435315 and rs7747345) on the risk of GDM were produced (Supplementary Figure S3). Additional methods yielded consistent results, including weighted mode (OR = 9.79, 95% CI = 5.05–18.99; *p* = 2.65 × 10^−5^), weighted median (OR = 6.02, 95% CI = 3.10–11.69, *p* = 1.12 × 10^−7^), and MR-Egger analysis (OR = 11.47, 95% CI = 2.56–51.42, *p* = 0.02) (Figure 3B). The results’ stability was further demonstrated by scatter plots (Figure 3C). 

**Figure 4:**
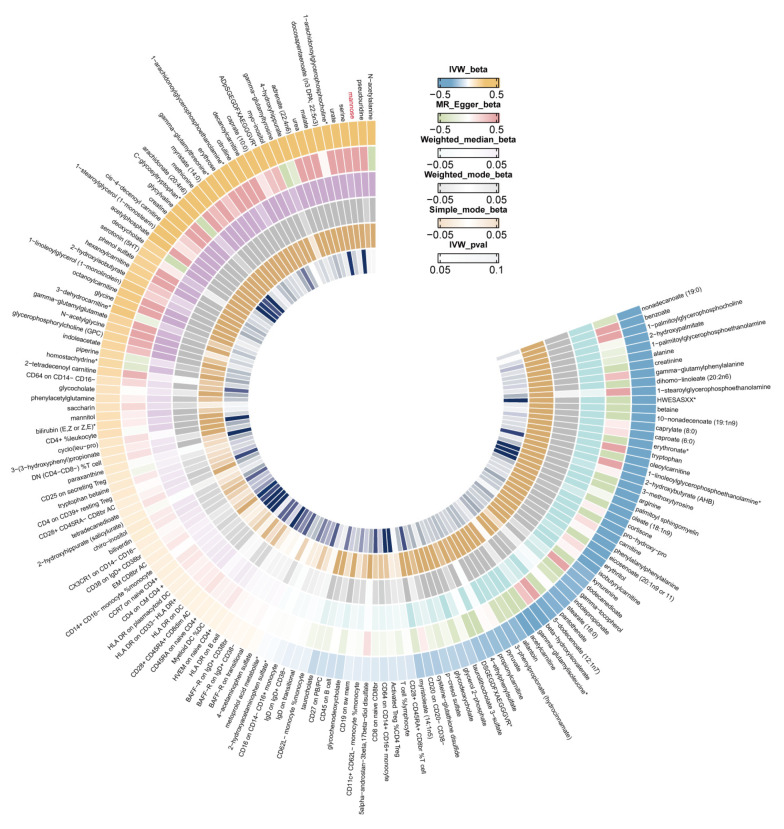
IVW (inverse-variance weighted) Mendelian randomization estimates for the associations between serum metabolites, immune traits, and gestational diabetes.

IVW estimates for the associations between serum metabolites, immune traits, and GDM are shown in Figure 4. The sensitivity analysis results for mannose on GDM are shown in Supplementary Figure S3. The test results from the MR-Egger (*p* = 0.37 > 0.05) and MR-PRESSO methods (*p* = 0.17 > 0.05) yielded *p*-values greater than 0.05, and the intercept of the MR-Egger regression was near 0 (<0.1), indicating an absence of evidence for horizontal pleiotropy and outlier variants (Supplementary Figure S3). According to the reverse MR analysis, there was no noticeable causal association between GDM and mannose, as shown in Supplementary Table S4. 

To ascertain whether the observed causal effect of mannose on GDM was a direct or indirect influence, we subsequently performed MVMR, adjusting for sex hormone-binding globulin levels (SHBG) [43], waist circumference [44], and cardiovascular disease (CVD) [45] (Supplementary Table S5). Multivariate analyses consistently demonstrated the persistent statistical significance of the association between mannose and GDM risk, further substantiated by corroborative findings from the forward univariable analysis. Furthermore, possible horizontal pleiotropy was not detected by the intercept term generated from MR-Egger.

**Figure 5:**
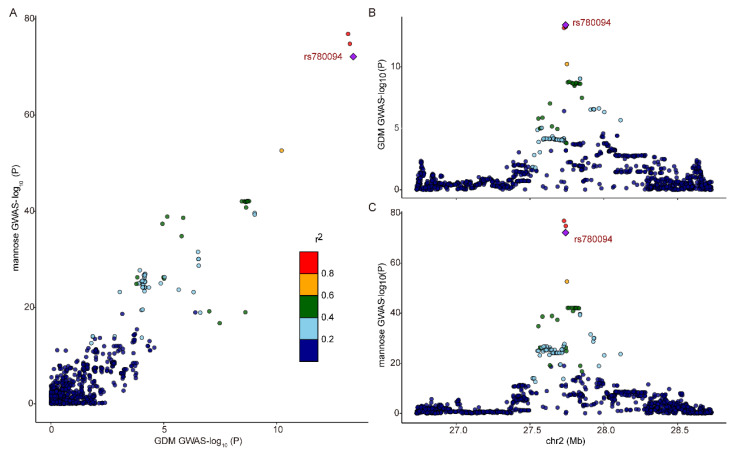
Colocalization analysis of mannose and gestational diabetes (GDM). Positive (PPH4 > 99%) colocalization results (**A**). When most points are located on the diagonal, it indicates GDM, GWAS, and mannose signals are likely colocalized. Variants are colored by their *R*^2^ value, and the risk variant is labeled and uniquely colored purple. SNP, single-nucleotide polymorphisms. −log10(*p*) association *p*-values for biomarker and −log10(*p*) association *p*-values for expression in GDM variants (**B**) and mannose SNPs (**C**), 1 Mb range.

Moreover, colocalization analysis revealed a higher posterior probability (PPH4 = 0.99), indicating that mannose and GDM shared a common causal signal within the 1 Mb locus surrounding rs1260326 (*p* = 1.30 × 10^−7^) (Figure 5). Collectively, the combination of MR and colocalization analysis reveals mannose may serve as a promising biomarker and therapeutic target for GDM.

### 3.4. Metabolic Pathway Analysis

**Figure 6:**
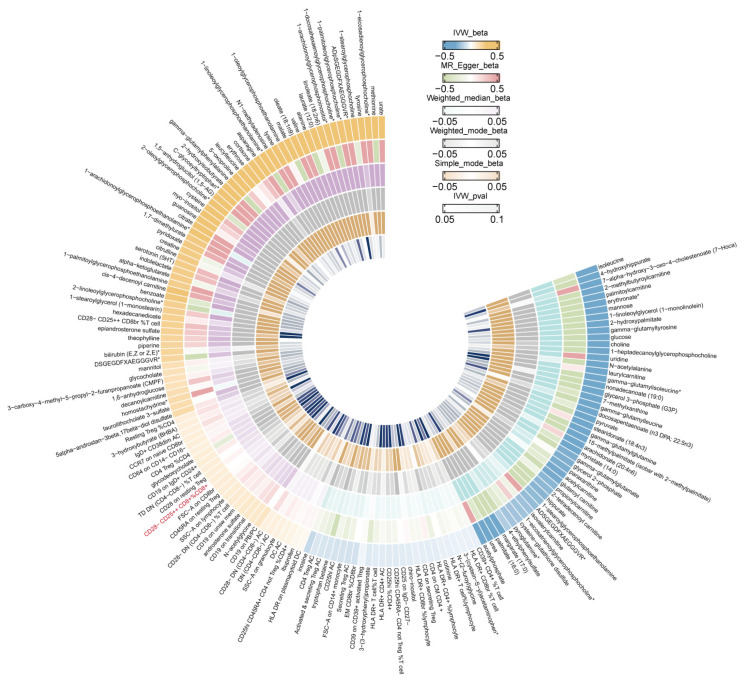
IVW (inverse-variance weighted) Mendelian randomization estimates for the associations between serum metabolites, immune traits, and female infertility.

The analysis of metabolic pathways revealed three significant pathways primarily associated with GDM and female infertility (Supplementary Table S6). Among these, ‘Arginine and proline metabolism’ appeared to be the most significantly implicated in the development of GDM (*p* = 0.000135). For female infertility, the most significant pathways were ‘Caffeine metabolism’ (*p* = 0.000277) and ‘Biosynthesis of unsaturated fatty acids’ (*p* = 0.00311). Moreover, IVW estimates for the associations between serum metabolites, immune traits, and infertility are shown in Figure 6.

## 4. Discussion

**Figure 7:**
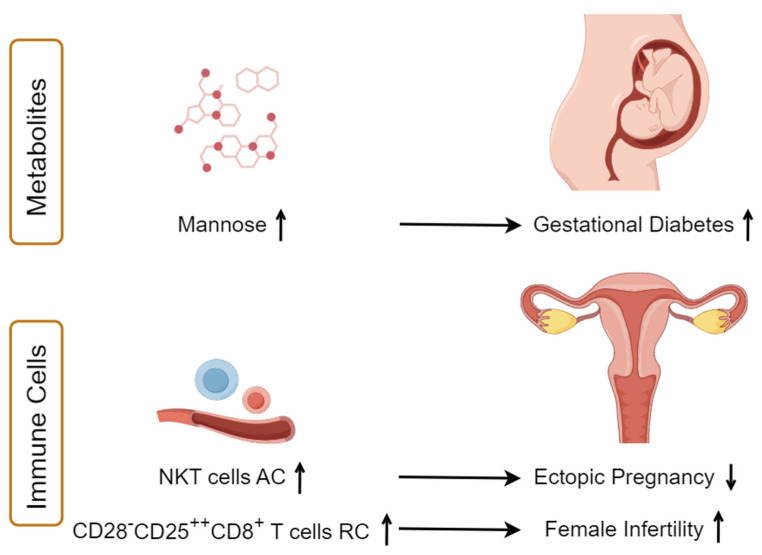
Summary of key findings. This figure illustrates the study’s main findings, showing high mannose levels linked to increased gestational diabetes risk, elevated CD28^−^CD25^++^CD8^+^ T cell relative counts (RC) associated with higher female infertility risk, and increased NKT cell absolute counts (AC) correlating with reduced ectopic pregnancy risk.

In this MR study, we harness the power of circulating biomarkers associated with both metabolism and immunity to investigate their potential causal roles in the development of 14 female reproductive diseases. Our results reveal mannose plays a causative role in the heightened risk of GDM. Furthermore, our study demonstrates a causal relationship between genetically predicted higher levels of circulating CD28^−^CD25^++^CD8^+^ T cells and an increased risk of female infertility. Conversely, an increased count of circulating NKT cells is linked to a lower risk of ectopic pregnancy (Figure 7). Rigorous sensitivity analyses affirm the reliability of our MR findings, along with the 95% CI for the effect excluding 0, indicating a statistically significant and potentially meaningful association. Despite the CI for the association between mannose and GDM being relatively wide (2.85–12.73), its clinically significant impact on maternal and offspring health underscores crucial implications for GDM prevention and management [46]. 

A growing number of studies have explored the genetic and metabolic basis of GDM, spurred by its rising prevalence and role in the global upswing of T2D. Strides in metabolomics have yielded valuable insights in comprehending the pathogenesis of GDM. For instance, Li et al. identified 36 serum metabolites potentially linked to GDM, including sterol lipids, fatty acyls, prenol lipids, sphingolipids, and glycerophospholipids, as detected using LC-MS [47]. Another recent study highlighted alterations in the metabolism of glycine, serine, arginine, and proline in GDM patients [48]. Unlike these observational studies, our MR approach, employing genetic variants as instrumental variables and leveraging GWAS summary statistics, revealed no direct causal link between GDM risk and lipid or amino acid metabolite levels. Crucially, a significant causal relationship was found between genetic variants, serum mannose levels, and GDM.

Mannose, a bioactive monosaccharide, is a key player in glycosylation processes and energy metabolism. Our findings align with prior research showing increased mannose levels in GDM patients’ fasting blood and amniotic fluid [49]. However, the earlier study fell short of establishing a direct causal relationship. Notably, elevated serum mannose levels strongly indicate future risk for various chronic diseases, such as PCOS [50], T2D [51], CVD [52], and albuminuria [51], potentially contributing to their development rather than serving solely as a novel biomarker. Several research studies illuminate the possible mechanisms behind mannose’s association with these diseases. According to recent research, circulating mannose levels are positively associated with obesity-independent insulin resistance due to mannose’s interference with insulin receptor function or its role in glycation end product [53]. Another potential pathway could be through the impact of mannose on the gut microbiome [54], further increasing GDM risk.

Dietary habits during pregnancy, particularly high sugar intake and low fruit and vegetable consumption, are also implicated in increased GDM risk [55]. This suggests that dietary modifications and mannose-targeted therapies could be effective GDM management strategies. Moreover, the impact of environmental factors [56,57,58] must be considered in the broader context of GDM etiology. Given these findings, future research should focus on larger sample sizes and more diverse populations to validate the observed relationships and to explore the underlying mechanisms. Such research is imperative for advancing the development of more effective preventative and therapeutic strategies for GDM and related metabolic disorders.

Female infertility affects 10% of women of reproductive age globally, resulting in a substantial decline in childbirth rates and contributing to significant population decreases [59]. A recent cross-sectional study found lower serum CD8 T cell levels in infertile patients compared to controls [60] and another identified T% and CD4+ T% as independent risk factors for PCOS [61]. Our study, utilizing genetic data, examined the causal relationship between circulating immune traits and infertility, thus minimizing biases from confounding factors. Contrasting with a previous study that found a significant association between epiandrosterone sulfate (EPIA-S) levels and PCOS [62], our stringent analysis did not establish a causal relationship between 486 serum metabolites, 731 immune cell traits, and PCOS.

Ectopic pregnancy is the leading cause of maternal morbidity and mortality rate with elusive etiology [63]. NKT cells, a unique subset of NK cells, are crucial in maintaining pregnancy and modulating immune system modulation during gestation [64]. Previous studies have shed light on the immune response in ectopic pregnancies. For instance, elevated CD69 staining and increased numbers of CD56^+^ and CD3^+^ cells were observed in ruptured ectopic pregnancies, highlighting the active participation of immune cells in this condition [65]. Moreover, the presence of Chlamydia trachomatis (anti-CT) Hsp60 immunity has been identified as a predominant feature in ectopic pregnancies [66]. Li et al. proposed that the joint detection of serum levels of progesterone, β-HCG, CA125, and the CD3^+^ T cell percentage could serve as reliable indicators for the early diagnosis of ectopic pregnancy [67]. However, the causal link between infectious agents and the resulting immune response and ectopic pregnancies has not been established previously. Our reverse MR analysis showed no potential reverse causal association between ectopic pregnancy and NKT cells AC. This suggests that the observed associations between NKT cells and ectopic pregnancy are likely unidirectional. As suggested by Li et al. [67], the investigation of new serum markers could lead to earlier diagnosis, timely medical treatment, and prevention. Thus, further research in larger prospective studies is imperative to unravel their implications for the early detection and management of the condition.

Our investigation has several innovations. Firstly, it utilizes peripheral blood immune cell signatures and metabolites as exposures, illuminating their potential causal links with reproductive diseases and underscoring profound implications for clinical research. Secondly, the adoption of a two-sample bidirectional MR approach minimizes the potential for confounders or reverse causality. The methods applied to evaluate the causal relationship between IVs and outcomes are valid, including IVW, weighted median, and MR-Egger. Thirdly, in contrast to preceding MR analyses involving single-exposure factors, the evaluation of 486 blood metabolites and 731 immune traits requires substantial computational resources and presents intricate analytical challenges. The analytical framework outlined in our study may serve as a valuable reference for analogous research endeavors.

A few drawbacks, however, exist in our study. Firstly, despite our rigorous application of various MR methodologies to address pleiotropy-induced confounding, the inherent nature of MR studies means that residual biases cannot be entirely eliminated. Secondly, the power of the IVs depends largely on the sample size of GWASs. Therefore, further research with larger and more diverse populations is crucial to reinforce the observed causal associations. Thirdly, MR studies often reveal the lifetime influence of risk variables on health outcomes, making it challenging to separate causes at different phases of the development of a disease. Consequently, additional research and mechanistic validations are essential to elucidate the roles of these factors in the pathogenesis of female reproductive disorders. 

## 5. Conclusions

In conclusion, our MR study provides new insights into the associations between serum metabolites, immune traits, and female reproductive disorders. We discovered a significant link between elevated mannose levels and increased susceptibility to GDM, suggesting its potential as a biomarker. Additionally, increased NKT cells AC were found to have a protective effect against ectopic pregnancy, while higher levels of circulating CD28^−^CD25^++^CD8^+^ T cells RC were causally associated with an increased risk of female infertility. These findings pave the way for developing early diagnostic tools and targeted treatments in reproductive health.

## Data Availability

Publicly available datasets were analyzed in this study. This data can be found here: (http://metabolomics.helmholtz-muenchen.de/gwas/, accessed on 1 July 2023) and (http://gwas.mrcieu.ac.uk/, accessed on 1 July 2023). Custom codes and preprocessed data are available on GitHub (https://github.com/DaXuanGarden/BioCycleMR, accessed on 2 October 2023). The datasets used and/or analyzed in the current study are available from the GWAS catalogue (https://www.ebi.ac.uk/gwas/summary-statistics, accessed on 1 July 2023).

## Supplementary Materials

The following supporting information can be downloaded at: https://www.mdpi.com/article/10.3390/biom14010116/s1, Table S1: The GWAS data source of 14 female reproductive diseases; Table S2: The most detrimental and protective immune cell traits factors for 14 female reproductive diseases; Table S3: The most detrimental and protective serum metabolites factors for 14 female reproductive diseases; Table S4: The reverse Mendelian randomization estimates between gestational diabetes and mannose; Table S5: The Multivariable Mendelian randomization estimates between gestational diabetes and mannose; Table S6: Metabolic pathway associated with gestational diabetes, and female infertility; Figure S1: Two-sample MR sensitivity analysis for serum immunophenotypes putatively causally associated with female infertility; Figure S2: Two-sample MR sensitivity analysis for serum immunophenotypes putatively causally associated with ectopic pregnancy; Figure S3: Funnel plot (A), forest plot (B), and leave-one-out plot (C) of the causa effect of mannose on gestational diabetes risk. IVW: Inverse-variance-weighted.

## Abbreviations

AC: absolute cell; CI: confidence interval; FBP: fructose-1,6-bisphosphate; GDM: gestational diabetes; GWAS: genome-wide association study; IV: instrumental variables; LD: linkage disequilibrium; IVW: inverse-variance weighted; MAF: middle allele frequency; MR: Mendelian randomization; MR-PRESSO: MR pleiotropy residual sum and outlier test; MFI: median fluorescence intensities; MP: morphological parameters; NKT: natural killer T cells; PVE: phenotypic variation explained; RC: relative cell; OR: odds ratio; SNP: single-nucleotide polymorphism; *R*^2^: explained variance.
