# Supplementary material for: Potential Causal Association between Plasma Metabolites, Immunophenotypes, and Female Reproductive Disorders: A Two-Sample Mendelian Randomization Analysis
Source: Biomolecules. 2024 Jan 16;14(1):116. doi: 10.3390/biom14010116 (PMC10813709; doi:10.3390/biom14010116)
Supplement: Supplementary file 1 [file biomolecules-14-00116-s001.zip › biomolecules-2784966-supplementary.pdf]

Supplementary files

Table S1 The GWAS data source of 14 female reproductive diseases.

Phenocode	Trait	num_cases	num_controls
E4_PCOS_BROAD	Polycystic ovarian syndrome, broad definition	3205	204492
GEST_DIABETES	Gestational diabetes (for exclusion)	13039	197831
N14_AMENORRHEAPRIM	Amenorrhoea	2220	107564
N14_ENDOMETRIOSIS	Endometriosis	15088	107564
N14_FEMALEINFERT	Female infertility	13142	107564
N14_UTER_POLYP	Uterine polyps	2871	107564
O15_ABORT_SPONTAN	Spontaneous abortion	16906	149622
O15_MEMBR_PREMAT_RUPT	Premature rupture of membranes	7147	168929
O15_PLAC_PRAEVIA	Placenta praevia	1232	168929
O15_POLYHYDR	Polyhydramnios	1209	168929
O15_POSTPART_HEAMORRH	Postpartum haemorrhage	8249	162777
O15_PREG_ECTOP	Ectopic pregnancy	5648	149622
O15_PRETERM	Preterm labour and delivery	8507	162777
O15_PRE_OR_ECLAMPSIA	Pre-eclampsia or eclampsia	7212	194266

Table S2 The most detrimental and protective immune cell traits factors for 14 female reproductive diseases

Outcome	Exposure	#SNPs	IVW		MR-Egger		Weighted median		Simple mode		Weighted mode	
			OR (95% CI)	P-value	OR (95% CI)	P-value	OR (95% CI)	P-value	OR (95% CI)	P-value	OR (95% CI)	P-value
Polycystic ovarian syndrome	CD28- CD25++ CD8+ %T cell	5	1.3393 (1.1180 - 1.6044)	0.001522	0.8726 (0.4839 - 1.5735)	0.681318	1.3827 (1.0954 - 1.7454)	0.006393	1.5197 (1.1138 - 2.0734)	0.057586	1.5182 (1.1067 - 2.0826)	0.060761
Polycystic ovarian syndrome	Naive DN (CD4-CD8-) AC	8	0.8359 (0.7150 - 0.9772)	0.024488	0.7556 (0.5370 - 1.0631)	0.158822	0.8202 (0.6702 - 1.0038)	0.054427	0.8786 (0.6557 - 1.1773)	0.414662	0.8088 (0.6248 - 1.0469)	0.150957
Gestational diabetes	CD4+ %leuko cyte	5	1.1627 (1.0421 - 1.2973)	0.006959	0.9442 (0.6864 - 1.2988)	0.747486	1.1792 (1.0181 - 1.3659)	0.027896	1.2097 (1.0090 - 1.4504)	0.10883	1.2139 (0.9903 - 1.4879)	0.135376
Gestational diabetes	CD20 on CD20- CD38-	6	0.8481 (0.7400 - 0.9719)	0.017829	1.1031 (0.8102 - 1.5019)	0.566774	0.8790 (0.7741 - 0.9980)	0.046561	0.7357 (0.5471 - 0.9893)	0.097933	0.9659 (0.8369 - 1.1147)	0.654867
Amenorrhoea	CD86 on monocyte	6	1.2680 (1.0947 - 1.4688)	0.001547	1.4037 (1.0416 - 1.8916)	0.089846	1.3103 (1.0964 - 1.5660)	0.002963	1.3290 (1.0852 - 1.6277)	0.040299	1.3228 (1.0588 - 1.6527)	0.05704
Amenorrhoea	CD28 on CD28+ DN (CD4-CD8-)	2	0.5423 (0.3211 - 0.9158)	0.022093	/	/	/	/	/	/	/	/

Endometriosis	CD3- lymphocyte % lymphocyte	3	1.1768 (1.0005 - 1.3842)	0.049344	0.1999 (0.0208 - 1.9193)	0.395914	1.1112 (0.9137 - 1.3513)	0.290806	1.0596 (0.7906 - 1.4202)	0.735844	1.0327 (0.7774 - 1.3719)	0.844675
Endometriosis	CD127- CD8+ %T cell	5	0.8711 (0.8053 - 0.9423)	0.000577	1.1265 (0.6158 - 2.0607)	0.724802	0.8853 (0.7977 - 0.9825)	0.021871	0.8167 (0.7037 - 0.9478)	0.056028	0.9237 (0.7947 - 1.0735)	0.35913
Female infertility	CD28- CD25++ CD8+ %T cell	5	1.2610 (1.1373 - 1.3982)	1.07E-05	1.4068 (0.9766 - 2.0265)	0.164215	1.2500 (1.0945 - 1.4276)	0.000996	1.3118 (1.0906 - 1.5779)	0.044988	1.2891 (1.0676 - 1.5564)	0.057565
Female infertility	EM CD8+ %CD8+	8	0.9100 (0.8289 - 0.9992)	0.047977	0.9901 (0.7823 - 1.2530)	0.936423	0.9107 (0.8054 - 1.0298)	0.135891	0.8959 (0.7664 - 1.0472)	0.20982	0.9005 (0.7941 - 1.0212)	0.146376
Uterine polyps	FSC-A on HLA DR+ T cell	6	1.3780 (1.1391 - 1.6670)	0.000963	1.8827 (0.9759 - 3.6321)	0.132189	1.3268 (1.0245 - 1.7184)	0.032098	1.2853 (0.8712 - 1.8961)	0.261585	1.3712 (0.9434 - 1.9931)	0.158929
Uterine polyps	PB/PC %lymphocyte	8	0.7941 (0.6696 - 0.9418)	0.008073	0.7325 (0.4544 - 1.1809)	0.248598	0.8333 (0.6729 - 1.0319)	0.09446	0.8183 (0.5924 - 1.1304)	0.263264	0.8931 (0.6778 - 1.1769)	0.448323
Spontaneous abortion	CD28- CD25++ CD8+ %T cell	5	1.1190 (1.0228 - 1.2242)	0.014209	1.3028 (0.9800 - 1.7319)	0.166185	1.0856 (0.9703 - 1.2146)	0.151465	1.0744 (0.9164 - 1.2597)	0.426515	1.0595 (0.9214 - 1.2184)	0.462604
Spontaneous abortion	CD62L- monocyte AC	7	0.9145 (0.8583 - 0.9743)	0.005708	0.8529 (0.6592 - 1.1036)	0.28028	0.9177 (0.8494 - 0.9915)	0.029594	0.9197 (0.8142 - 1.0389)	0.226659	0.9138 (0.8220 - 1.0159)	0.146182

Premature rupture of membranes	CD25 on activated Treg	7	1.2666 (1.0251 - 1.5649)	0.028554	1.1102 (0.3526 - 3.4954)	0.865267	1.4037 (1.1371 - 1.7327)	0.001601	1.4940 (1.1297 - 1.9757)	0.030539	1.4303 (1.1435 - 1.7889)	0.020197
Premature rupture of membranes	CD8 on CM CD8+	8	0.8665 (0.8053 - 0.9324)	0.000126	0.8777 (0.7116 - 1.0827)	0.268946	0.8591 (0.7776 - 0.9491)	0.002815	0.8427 (0.7180 - 0.9889)	0.074265	0.8559 (0.7532 - 0.9726)	0.048389
Placenta praevia	CD25 on activated Treg	7	1.8835 (1.2288 - 2.8869)	0.003667	1.4314 (0.1496 - 13.6916)	0.768139	1.4478 (0.8714 - 2.4054)	0.153124	1.2593 (0.4691 - 3.3810)	0.663358	1.0489 (0.4183 - 2.6307)	0.922191
Placenta praevia	CD62L on granulocyte	8	0.7271 (0.5629 - 0.9391)	0.01464	0.5802 (0.2807 - 1.1989)	0.191944	0.6927 (0.4956 - 0.9683)	0.031665	0.6756 (0.4176 - 1.0932)	0.154313	0.6756 (0.4405 - 1.0363)	0.115466
Polyhydramnios	CD39+ activated Treg %activated Treg	2	1.6648 (1.1009 - 2.5175)	0.015705	/	/	/	/	/	/	/	/
Polyhydramnios	IgD- CD38- %B cell	7	0.7331 (0.5535 - 0.9711)	0.030407	0.7044 (0.2950 - 1.6819)	0.465829	0.6784 (0.4781 - 0.9627)	0.02979	0.6196 (0.3718 - 1.0326)	0.115858	0.6368 (0.3886 - 1.0436)	0.123513
Postpartum haemorrhage	TCRgd %T cell	8	1.1663 (1.0530 - 1.2917)	0.003158	1.1885 (0.9253 - 1.5264)	0.22506	1.1628 (1.0190 - 1.3269)	0.025123	1.1619 (0.9645 - 1.3996)	0.158172	1.1619 (0.9705 - 1.3910)	0.146347
Postpartum haemorrhage	Lymphocyte AC	6	0.8519 (0.7722 - 0.9398)	0.001384	0.9683 (0.7187 - 1.3047)	0.842837	0.8498 (0.7422 - 0.9731)	0.018528	0.8140 (0.6672 - 0.9931)	0.098294	0.8380 (0.6970 - 1.0074)	0.118659

Pre-eclampsia or eclampsia	BAFF-R on IgD- CD38br	9	1.1489 (1.0532 - 1.2533)	0.001761	1.1436 (0.9578 - 1.3654)	0.181451	1.1589 (1.0445 - 1.2859)	0.005405	0.9859 (0.8058 - 1.2063)	0.893802	1.1704 (1.0483 - 1.3067)	0.023236
Pre-eclampsia or eclampsia	CD45RA on CD39+ resting Treg	4	0.8032 (0.6875 - 0.9383)	0.005738	0.8522 (0.6355 - 1.1427)	0.397083	0.7890 (0.6479 - 0.9608)	0.018378	0.7513 (0.5892 - 0.9578)	0.104244	0.7907 (0.6392 - 0.9780)	0.11896
Ectopic pregnancy	CD27 on CD20-	5	1.2440 (1.0702 - 1.4459)	0.004452	1.1692 (0.8437 - 1.6203)	0.416933	1.2604 (1.0351 - 1.5346)	0.021242	1.3092 (1.0117 - 1.6942)	0.109863	1.2983 (0.9963 - 1.6920)	0.12545
Ectopic pregnancy	CD127 on CD45RA- CD4 not Treg	7	0.8661 (0.7549 - 0.9936)	0.040163	0.7418 (0.4366 - 1.2602)	0.319559	0.8381 (0.7055 - 0.9956)	0.044409	0.8907 (0.7109 - 1.1159)	0.353054	0.8525 (0.6982 - 1.0408)	0.168179
Preterm labour and delivery	IgD- CD38br AC	6	1.1662 (1.0235 - 1.3287)	0.020967	1.1278 (0.7220 - 1.7618)	0.625009	1.2115 (1.0550 - 1.3913)	0.006545	1.2416 (0.9718 - 1.5862)	0.143936	1.2393 (0.9626 - 1.5954)	0.156891
Preterm labour and delivery	TD CD8+ %CD8+	7	0.8555 (0.7338 - 0.9974)	0.046187	1.6280 (0.8617 - 3.0757)	0.193539	0.8964 (0.7769 - 1.0342)	0.133856	0.8378 (0.6442 - 1.0895)	0.234777	0.8543 (0.7049 - 1.0353)	0.159303

Table S3 The most detrimental and protective serum metabolites factors for 14 female reproductive diseases

Outcome	Exposure	#SNPs	IVW		MR-Egger		Weighted median	
			OR (95% CI)	P-value	OR (95% CI)	P-value	OR (95% CI)	P-value
Polycystic ovarian syndrome	X-06267	7	10.3248 (1.7706 - 60.2047)	0.009456	0.0351 (0.0000 - 31.0449)	0.377647	3.2667 (0.3242 - 32.9179)	0.315234
Polycystic ovarian syndrome	X-11381	9	0.1384 (0.0377 - 0.5073)	0.002844	0.0963 (0.0026 - 3.5170)	0.243037	0.1341 (0.0235 - 0.7659)	0.023819
Gestational diabetes	mannose	8	6.0248 (2.8507 - 12.7329)	2.55E-06	11.4653 (2.5566 - 51.4177)	0.018932	6.0233 (3.1028 - 11.6929)	1.12E-07
Gestational diabetes	arginine	10	0.3545 (0.1762 - 0.7131)	0.003636	0.2407 (0.0275 - 2.1036)	0.233867	0.5443 (0.2111 - 1.4035)	0.208146
Amenorrhoea	dihomo-linoleate (20:2n6)	3	12.1715 (1.2181 - 121.6220)	0.033338	1032.9179 (0.0305 - 34990585.7106)	0.416453	17.8544 (0.9687 - 329.0687)	0.052546
Amenorrhoea	tryptophan	108	0.1466 (0.0345 - 0.6235)	0.009333	20997.8440 (1.0266 - 429469121.3075)	0.052009	0.1964 (0.0292 - 1.3225)	0.094376

Endometriosis	C-glycosyltryptophan*	14	3.3564 (1.5124 - 7.4489)	0.002909	0.380821856	0.380822	2.8923 (0.9546 - 8.7627)	0.060391
Endometriosis	margarate (17:0)	5	0.3512 (0.1770 - 0.6968)	0.00276	0.6209 (0.0469 - 8.2161)	0.741534	0.3545 (0.1524 - 0.8246)	0.016054
Female infertility	1-oleoylglycerophosphoethanolamine	4	2.8987 (1.1204 - 7.4992)	0.028201	1.0499 (0.0022 - 506.3813)	0.989087	1.4278 (0.5425 - 3.7580)	0.470691
Female infertility	gamma-glutamylisoleucine*	15	0.5069 (0.3187 - 0.8062)	0.00411	0.4984 (0.1429 - 1.7383)	0.294408	0.5260 (0.2710 - 1.0210)	0.057609
Uterine polyps	X-06226	15	8.7176 (2.1110 - 35.9996)	0.002766	1.4909 (0.0738 - 30.1216)	0.798629	6.4739 (0.8330 - 50.3117)	0.0742
Uterine polyps	oleoylcarnitine	4	0.1338 (0.0306 - 0.5857)	0.007578	0.4295 (0.0000 - 57586.4350)	0.901269	0.1014 (0.0162 - 0.6339)	0.014382
Spontaneous abortion	X-06226	15	2.2842 (1.1748 - 4.4412)	0.014893	2.0188 (0.4527 - 9.0034)	0.373861	2.0172 (0.8533 - 4.7687)	0.109911
Spontaneous abortion	gamma-glutamylleucine	17	0.5486 (0.3210 - 0.9375)	0.028083	0.7608 (0.2552 - 2.2676)	0.630724	0.4790 (0.2276 - 1.0082)	0.052571

Premature rupture of membranes	X-03003	3	10.1786 (1.4554 - 71.1855)	0.019378	52000.7668 (0.0000 - 2212493715523889444301470894653440.0000)	0.801181	8.1395 (0.7363 - 89.9764)	0.087208
Premature rupture of membranes	creatinine	14	0.0898 (0.0212 - 0.3809)	0.001077	0.0941 (0.0033 - 2.6517)	0.190518	0.0824 (0.0121 - 0.5619)	0.01082
Placenta praevia	X-06351	4	12.2966 (1.5915 - 95.0059)	0.016151	6.6427 (0.1844 - 239.3296)	0.409253	9.2314 (0.6223 - 136.9510)	0.106261
Placenta praevia	threonine	13	0.0174 (0.0018 - 0.1699)	0.000494	0.5807 (0.0002 - 1691.1076)	0.896179	0.0036 (0.0002 - 0.0716)	0.000223
Polyhydramnios	linoleate (18:2n6)	9	15.3591 (1.4681 - 160.6866)	0.022575	477.1073 (0.0158 - 14410875.8305)	0.279584	17.5607 (0.8996 - 342.7796)	0.058726
Polyhydramnios	acetylcarnitine	7	0.1135 (0.0212 - 0.6068)	0.010952	0.1799 (0.0027 - 11.8784)	0.458774	0.2421 (0.0263 - 2.2241)	0.209978
Postpartum haemorrhage	dihomo-linoleate (20:2n6)	3	3.6961 (1.0936 - 12.4917)	0.035378	3.5148 (0.0142 - 869.4523)	0.73236	2.7386 (0.5745 - 13.0556)	0.206114
Postpartum haemorrhage	caproate (6:0)	17	0.3308 (0.1330 - 0.8231)	0.017375	1.7842 (0.1004 - 31.7050)	0.698876	0.6209 (0.1921 - 2.0064)	0.425776

Pre-eclampsia or eclampsia	histidine	6	13.5240 (1.6825 - 108.7046)	0.014313	13.5446 (0.1921 - 954.9804)	0.296279	7.2341 (0.4925 - 106.2630)	0.148918
Pre-eclampsia or eclampsia	X-05907	9	0.2085 (0.0889 - 0.4888)	0.00031	0.1049 (0.0135 - 0.8137)	0.067884	0.1401 (0.0456 - 0.4302)	0.000595
Ectopic pregnancy	lactate	5	5.8178 (1.4892 - 22.7275)	0.011314	5.7958 (1.0638 - 31.5778)	0.042208	3.8711 (0.0486 - 308.2990)	0.587269
Ectopic pregnancy	uridine	15	0.1006 (0.0244 - 0.4153)	0.0015	0.2273 (0.0015 - 35.5033)	0.575201	0.1059 (0.0141 - 0.7970)	0.029231
Preterm labour and delivery	X-11497	4	3.9472 (1.2540 - 12.4242)	0.018929	18.7545 (0.0376 - 9359.5864)	0.423274	5.7676 (1.4110 - 23.5755)	0.014715
Preterm labour and delivery	X-04494	8	0.3519 (0.1508 - 0.8211)	0.015693	0.5752 (0.0448 - 7.3915)	0.686021	0.3742 (0.1199 - 1.1677)	0.090464

Table S4 The reverse Mendelian randomization estimates between gestational diabetes and mannose.

id.outcome	outcome	exposure	method	nsnp	b	se	pval
met-a-314	Mannose	Gestational diabetes	MR Egger	9	-0.04726	0.070646	0.524919
met-a-314	Mannose	Gestational diabetes	Weighted median	9	0.011714	0.00737	0.111962
met-a-314	Mannose	Gestational diabetes	Inverse variance weighted	9	0.039128	0.035129	0.265342
met-a-314	Mannose	Gestational diabetes	Simple mode	9	0.013097	0.010788	0.25938
met-a-314	Mannose	Gestational diabetes	Weighted mode	9	0.011161	0.007022	0.150649
ebi-a-GCST90001621	Natural Killer T Absolute Count	Ectopic pregnancy	Wald ratio	1	-0.06834	0.251239	0.785605
ebi-a-GCST90001676	CD28- CD25++ CD8+ T cell %T cell	Female infertility	Wald ratio	1	-0.13711	0.540848	0.799878

Table S5 The Multivariable Mendelian randomization estimates between gestational diabetes and mannose.

id.exposure	exposure	outcome	b	se	pval	lo_ci	up_ci	OR	OR_lci95	OR_uci95
ebi-a-GCST90025958	Sex hormone-binding globulin levels	Gestational diabetes	-0.1710738	0.051223	0.000838	-0.27147	-0.07068	0.842759	0.762257	0.931763
ebi-a-GCST90038595	Cardiovascular disease	Gestational diabetes	1.10775915	0.461355	0.016346	0.203504	2.012014	3.027566	1.22569	7.478366
ebi-a-GCST90103753	Waist circumference	Gestational diabetes	-0.047342	0.086803	0.58548	-0.21748	0.122791	0.953761	0.804548	1.130648
met-a-314	Mannose	Gestational diabetes	2.22987727	0.398521	2.2E-08	1.448776	3.010979	9.298725	4.2579	20.30726

The Univariable Mendelian randomization estimates between exposures and gestational diabetes.

id.exposure	adjustment	methods	b	se	pval	lo_ci	up_ci	or	or_lci95	or_uci95
ebi-a-GCST90025958	Sex hormone-binding globulin levels	MR Egger	-0.01257214	0.071657	0.86077	-0.15302	0.127875	0.987507	0.858113	1.136411
		Weighted median	-0.14934815	0.052247	0.004256	-0.25175	-0.04694	0.861269	0.777438	0.95414

		Inverse variance weighted	-0.16377379	0.042821	0.000131	-0.2477	-0.07984	0.848934	0.780591	0.923261
		Simple mode	-0.15610447	0.136315	0.252471	-0.42328	0.111073	0.85547	0.654894	1.117477
		Weighted mode	-0.07216286	0.059051	0.222045	-0.1879	0.043578	0.930379	0.828695	1.044541
ebi-a-GCST90038595	Cardiovascular disease	MR Egger	-0.04185903	0.415182	0.919739	-0.85562	0.771898	0.959005	0.425021	2.16387
		Weighted median	0.585550042	0.173278	0.000727	0.245924	0.925176	1.795979	1.278803	2.522312
		Inverse variance weighted	0.65913162	0.155875	2.35E-05	0.353617	0.964647	1.933113	1.424209	2.62386
		Simple mode	0.371465282	0.549011	0.49901	-0.7046	1.447527	1.449858	0.494308	4.252586
		Weighted mode	0.719090268	0.393633	0.068407	-0.05243	1.490612	2.052565	0.94892	4.439811
ebi-a-GCST90103753	Waist circumference	MR Egger	0.365127856	0.516813	0.499933	-0.64782	1.37808	1.440698	0.523183	3.967279

	Weighted median	0.159880032	0.142614	0.262257	-0.11964	0.439403	1.17337	0.887238	1.55178
	Inverse variance weighted	0.188786715	0.134957	0.161855	-0.07573	0.453303	1.207783	0.927067	1.573501
	Simple mode	-0.25416308	0.339816	0.473584	-0.9202	0.411876	0.775565	0.398438	1.509647
	Weighted mode	0.50162892	0.321723	0.15338	-0.12895	1.132205	1.651409	0.87902	3.102491

Table S6 Metabolic Pathway Associated with gestational diabetes, and female infertility.

Gestational diabetes

Metabolic Pathway	P-Value	FDR
Arginine and proline metabolism	0.000135	0.0114
Arginine biosynthesis	0.0448	1
Fructose and mannose metabolism	0.0635	1
Galactose metabolism	0.085	1
Glycine, serine and threonine metabolism	0.103	1
Amino sugar and nucleotide sugar metabolism	0.115	1
Fatty acid biosynthesis	0.144	1
Aminoacyl-tRNA biosynthesis	0.147	1

Female infertility

Metabolic Pathway	P-Value	FDR
Caffeine metabolism	0.000227	0.0191
Biosynthesis of unsaturated fatty acids	0.00311	0.131
Fatty acid elongation	0.0954	1
Fatty acid degradation	0.0979	1
Fatty acid biosynthesis	0.117	1

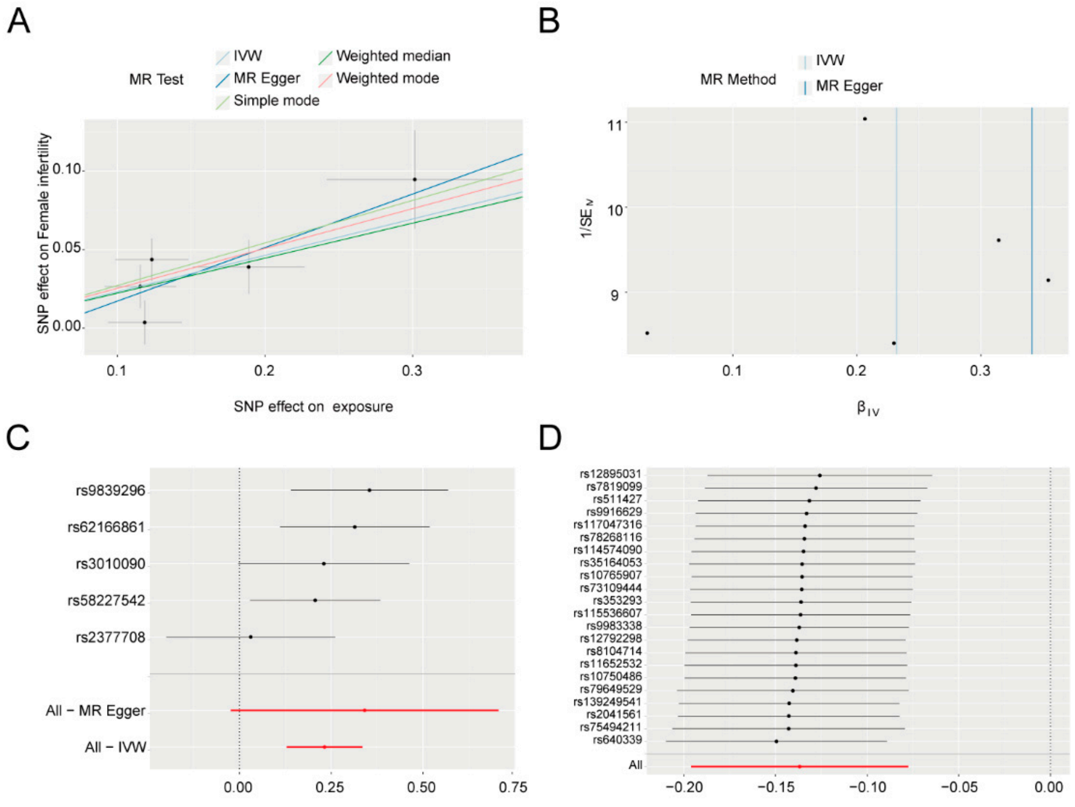

Figure S1. Scatter plot (A), funnel plot (B), forest plot (C), and leave-one-out plot (D) of the causa effect of CD28$^-$CD25^{++}CD8$^+$ T cells relative count and female infertility risk. IVW: Inverse-variance-weighted; SNP: single-nucleotide polymorphism

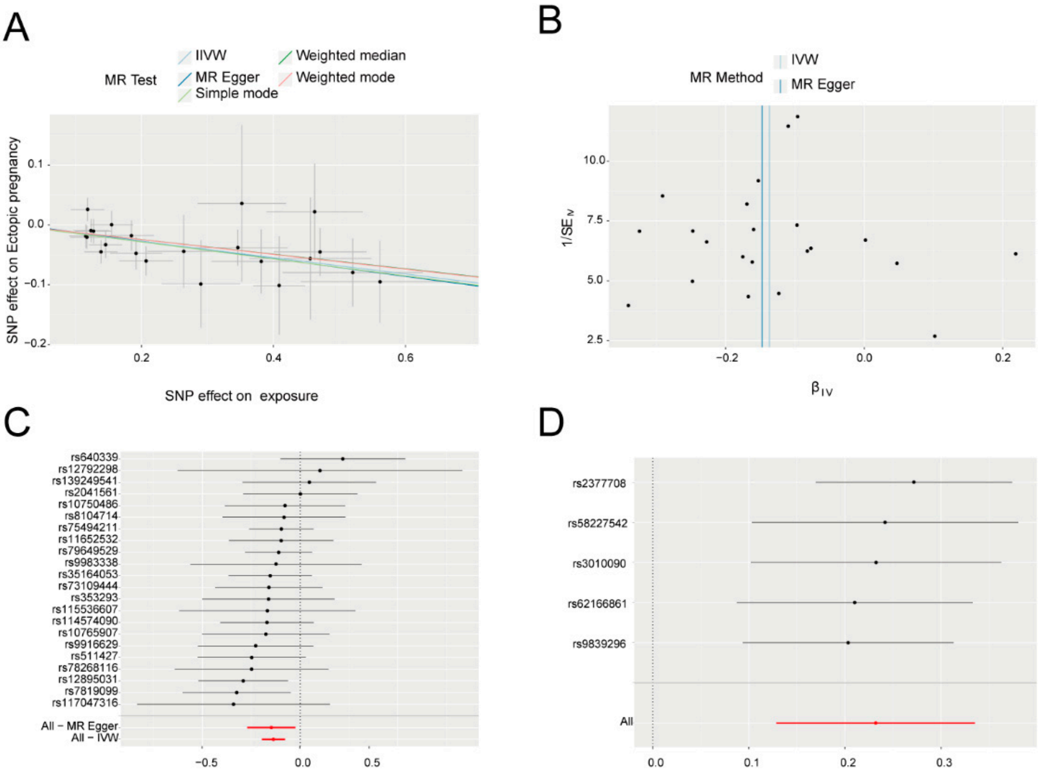

Figure S2. Scatter plot (A), funnel plot (B), forest plot (C), and leave-one-out plot (D) of the causa effect of NKT cells absolute count on ectopic pregnancy risk. IVW: Inverse-variance-weighted; SNP: single-nucleotide polymorphism

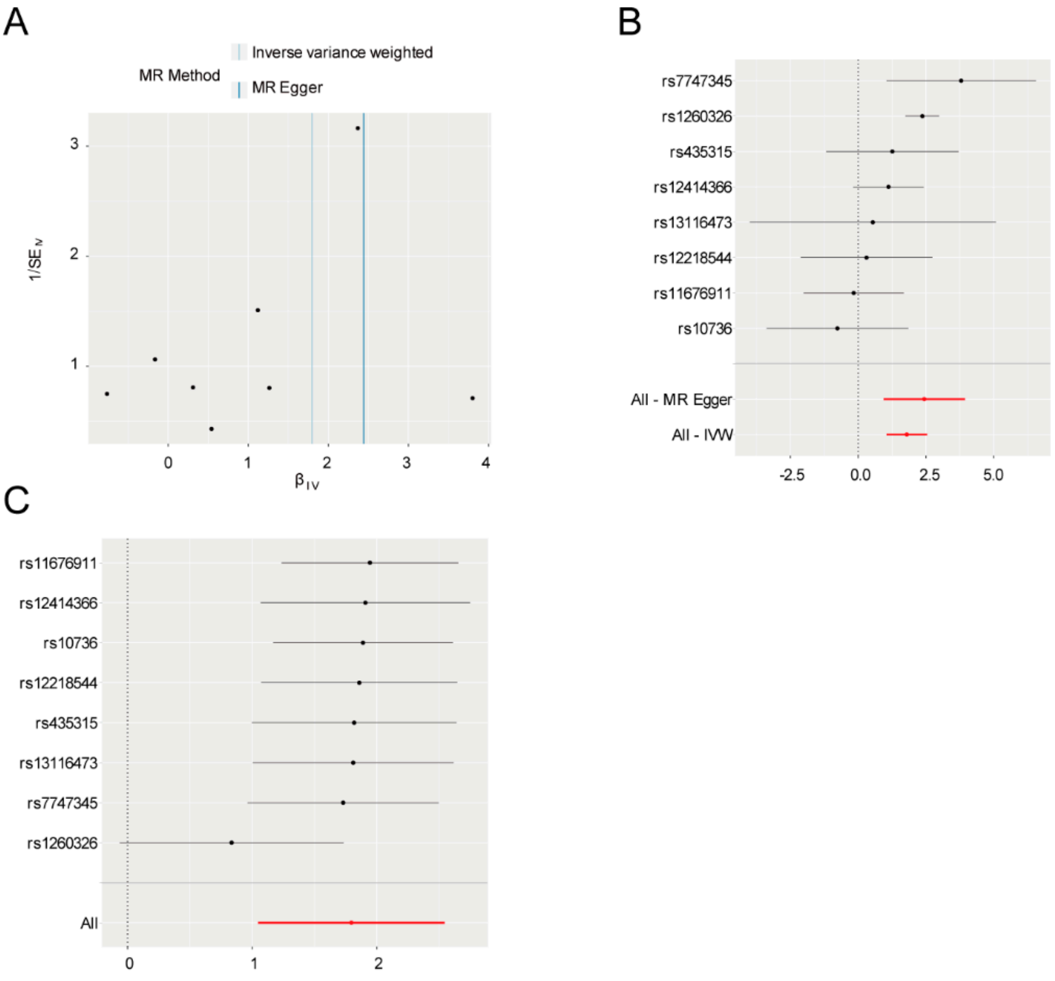

Figure S3. Funnel plot (A), forest plot (B), and leave-one-out plot (C) of the causa effect of mannose on gestational diabetes risk. IVW: Inverse-variance-weighted
